# A Phase-Dependent Effect That Enables Multi-Scale Moisture Measurements in Heterogeneous Substrates Using Tubular *RH* Sensors

**DOI:** 10.3390/s22103887

**Published:** 2022-05-20

**Authors:** Detlef Lazik

**Affiliations:** Department Soil System Science, Helmholtz Centre for Environmental Research GmbH—UFZ, 06120 Halle (Saale), Germany; detlef.lazik@ufz.de

**Keywords:** soil, fertility, soil litter, ignition risk, water saturation, heterogeneity, water sorption, membrane, sensor

## Abstract

A knowledge of the moisture in soils/soil litter allows for the estimation of irrigation needs or the risk of forest fire. A membrane-based humidity sensor (MHS) can measure the relative humidity (RH) as an average value in such heterogeneous substrates via its sensitive tubular silicone membrane. This RH corresponds to the moisture-dependent water potential of the substrate. For humid conditions in soil, however, the RH is already larger than 98% and hence is insensitively correlated with the water potential. For such conditions, a step-like response of the MHS was found, which occurs if the silicone membrane is wetted with water. This appears to correspond to oversaturated water vapor and must be attributed to a phase-dependent sorption mechanism of the membrane. This effect allows the expansion of the range of applications of the MHS in the detection of liquid water, such as in dew point detection. Based on this, the dependency of the measurement signal on the mean water saturation in a substrate along the tubular membrane is demonstrated. A comparison of the measurement signal with an internal reference signal according to the MHS measurement principle makes it possible to distinguish this new, saturation-dependent measurement scale from the one used for RH measurement.

## 1. Introduction

Relative humidity RH [%] is one of the most frequently measured parameters and is required in various sectors of society and environmental monitoring. The demand for humidity sensors and the variety of products offered are therefore large [1]. Until now, commercially available humidity sensors have yielded comparatively large measurement errors for high RH, i.e., close to the saturation of water vapor (referred to simply as ‘vapor’). Furthermore, the condensation or absorption of water onto or within the humidity sensor can change its detection behavior or even damage it. However, in this region of high RH, there are important potential applications for humidity measurements, e.g., in soil and soil litter (referred to here as ‘litter’), as shown in the following two examples.

(i) The RH can enable us to determine the vapor activity in soil, which is the gas phase equivalent of the water potential [2,3] of liquid soil water. In dry soils, water movement in the gas phase contributes significantly to the soil water balance, but estimates of the magnitude of this term vary [4,5,6,7,8,9,10,11,12]. Driven by the expansion of drylands, the interest in considering the water movement in dry soils (see the review in [13]) and vapor fluxes (e.g., in numerical models) [14,15,16] has increased. When the RH is between 89.0% and 98.5%, soil contains liquid water only in the form of thin films that cover the solid surfaces. For a value of RH above 99%, capillary water is present, which is available to most plants, meaning that the soil becomes fertile. Even if commercially available humidity sensors are sufficiently capable of resolving the relatively narrow range of RH (≈1%) that is relevant to most plants, they generally only allow for point-based measurement, i.e., they represent the RH level at a local scale, meaning that the informative value for systems that are spatially highly heterogeneous, such as soils, is limited. The same applies to a large number of the measuring systems used in practice to determine the soil moisture [17,18,19,20,21,22]. In particular, a field study for direct determination of water content and water potential (or matrix potential) confirms this statement for different soil water sensors used in agricultural practice [18]. The sensors used for the study (in Table 1 in [18]) had a probe length of 20 cm maximum. A recent review summarized the situation as follows: “The measurement of soil moisture in agriculture is currently dominated by a small number of sensors, the use of which is greatly limited by their small sampling volume, high cost, need for close soil–sensor contact, and poor performance in saline, vertic and stony soils.” (Hardie, p. 1 in [23]).

(ii) If the water content near the soil surface decreases, such as in the litter layer of a temperate forest, the ignition risk of the litter will rise. As long as liquid water is still available, a high value of RH can be assumed, depending on the meteorological conditions (radiation, temperature, and wind), for the properties of the litter (layer height, density, and type of litter) and the measurement depth within this layer. When the liquid water phase has evaporated, the heat energy supplied is no longer compensated by the latent heat of the water. Hence, the temperature increases, the relative humidity decreases, and the risk of ignition is heightened: “The moisture content is considered to be the most critical factor that affects the ignitability of vegetation […] It also impacts fire propagation […] Therefore, it becomes an essential indicator in fire risk analysis” (Dahanayake and Chow, pp. 127–128 in [24]). Therefore, measuring moisture in the litter layer could be useful as a predictive indicator for fire risk assessment if the measured value adequately accounts for the heterogeneity of the litter layer. 

There may therefore be interest in a robust sensor that (i) enables an analysis of the moisture over a large range (ii) in a harsh environment (such as soil, litter, etc.) and (iii) yields a representative mean over the heterogeneity of this substrate (iv) over a long period without the need to periodically calibrate the sensor. A membrane-based humidity sensor (MHS) [25] that works based on the selective diffusion of gases through tubular membranes should be capable of measuring the average relative humidity over an area on the order of $10^{0}$ to $10^{3}$ m^2^ [26,27,28]. We demonstrated a suitable measurement range of up to nearly 100% for the MHS in our prior experiments and therefore believe that the MHS could function well in dry soils [3], i.e., below the vapor saturation of the soil air. The response of the MHS in the presence of a liquid water phase, however, has not previously been investigated. 

This study considers the response of the MHS in a vapor-saturated environment with changes of the aggregate state of water around the outer surface of the sensor. For saturated vapor, for example, above a flat water surface, it is generally assumed that both phases should have the same chemical potential (phase equilibrium), which should result in equivalent water potentials [29], with a water activity of $a_{w}=1$, defined by $a_{w}=RH/100\%$. For such a phase equilibrium, the presence of liquid water or vapor at the outer surface of the sensor membrane is not expected to affect the measurement; nevertheless, such an effect was observed. Based on the assumption that the water permeability in the membrane wall of the sensor cannot be influenced by the outer phase conditions, a phase-dependent boundary layer must exist. In this study, this boundary layer is investigated for vapor saturation, that is, for the corresponding saturation vapor pressure $e_{w}^{\prime }$ [hPa] of water in air that was calculated using the Magnus formula [30]:

(1) $$e_{w}^{\prime }=g\;(p_{air})\cdot a\cdot \exp\left(\frac{f\vartheta }{h+\vartheta }\right),\;g\;(p_{air})=1.0016+3.15\cdot 10^{-6}p_{air}-0.074\;p_{air}{}^{-1},$$

where ϑ [°C] is the temperature, $p_{air}$ [hPa] is the air pressure, and the parameters are a=6.112 hPa, $f=17.62\;{}^{{}^{\circ}}C^{-1}$, and $h=243.12\;{}^{{}^{\circ}}C$.

Although the MHS was originally designed to measure RH, this investigation reveals that this MHS also allows the detection of the presence or absence of liquid water, which is normally not possible via an RH measurement.

## 2. Basics

### 2.1. Measurement Principle of an MHS

**Figure 1:**
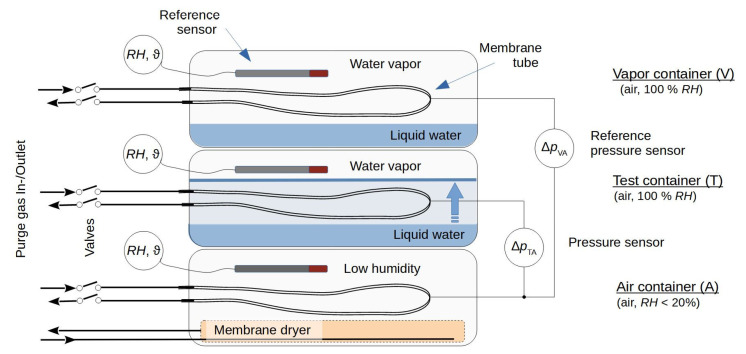
The setup consists of closed containers that enable the membrane tubes of the MHS to be placed in different environments: dry air (air container), vapor-saturated air (vapor container) and vapor-saturated air or liquid water (test container). The membrane tubes are connected via gas-tight tubes to valves and pressure sensors to allow the determination of the pressure differences $\Delta p_{\mathrm{TA}}$ and $\Delta p_{\mathrm{VA}}$ between the chambers connected in this way. Sensors for the relative humidity (RH) and temperature (ϑ) are placed in the upper parts of the containers.

An ideal MHS consists of three closable membrane tubes (as illustrated in Figure 1), in which the gas pressures are measured in a measurement step, which were purged with a gas of known composition (air) in a previous conditioning step. However, the construction of a real MHS involves additional components that influence its response. Therefore, to enable a consistent description, the following terms were used: a “cell” is an individual measurement cell of the MHS and consists of an air-filled measurement chamber called a “chamber”, the valves required to close this chamber, a pressure sensor for measuring the gas pressure inside it, and the connecting tubes and fittings. The chamber mainly consists of the inner volume of the gas-selective membrane tube, but the additional components (connecting tubes, fittings, etc.) form an additional dead volume that needs to be considered in the context of this investigation. The conditioning step allows for the adjustment of the steady-state diffusive gas flow through the wall of the membrane via purging with air through the opened chamber. In the subsequent measurement step, the gas diffusion through the membrane wall leads to changes in the partial pressure in the (now closed) chamber, which result in a pressure change, ∝ =dp/dt [hPa/s] (where p [hPa] is the gas pressure, and t [s] is the time). Near the steady state, the pressure change depends linearly on the differences between the concentrations (partial pressures) of the air components inside and outside the chamber. The MHS compares the pressure change for air with an unknown relative humidity, $RH_{x}$, with the pressure change for the vapor-saturated air. If the RH for the comparatively dry purge gas (air) is known, these two reference states are sufficient to determine $RH_{x}$. Silicone (PDMS—polydimethylsiloxane) tubes are used as the gas-permeable walls of the chambers. The gas transport properties of this non-porous rubber have been characterized in various studies [31,32,33,34,35,36]. PDMS has a high selectivity for H_2_O compared to other atmospheric gases, such as N_2_, O_2_, Ar, and CH_4_ [25]. It is an order of magnitude more selective for water than it is for CO_2_, i.e., only for a substantial CO_2_ concentration or a low temperature would a different membrane material be needed for the RH measurement.

### 2.2. Experimental Setup

The experimental setup (Figure 1) consists of three closed 5.6 l plastic containers stacked on top of each other. The air container (“A”) at the bottom was prepared to hold relatively dry air around a membrane tube, and a vapor container (“V”) was placed at the top to adjust the vapor saturation around a second membrane tube. A test container (“T”), located between the other two containers, was prepared to allow the vapor-saturated air to be displaced by liquid around a third membrane tube during the experiment. The air container was equipped with a membrane dryer made from a PDMS tube (length: 4.5 m, inner diameter: 9 mm, outer diameter: 11 mm), which was connected upstream to dried compressor air and downstream to the air in the laboratory. The test and vapor containers were equipped with tubes to enable water to be added to them. To achieve pressure equilibration with the outer air pressure, the air-filled inner space of each container was connected via a syringe filter (pore size 0.2 µm, filter diameter 4 mm) to the ambient air. For better equilibration of the temperature between the containers, the stack of containers was covered with cloths.

The membrane tube in the test container was placed between horizontally arranged plastic grids that were used to define its position as the water level rose above or fell below these grids. The membrane tubes in the air and vapor containers were set up in the same way using similar plastic grids. The grids had wide meshes of size 60 mm × 7 mm and a web thickness of 3 mm to ensure that most of the tube surface was not in contact with these supports. Two different membrane tube sets were used to construct the cells, as follows:

Set 1: $V_{i}=r_{i}\times r_{o}\times L=1.6\;\mathrm{mm}\times 2.4\;\mathrm{mm}\times 1000\;\mathrm{mm}$ (silicone peroxide, Fisher Bioblock Scientific, Illkirch, France),
Set 2: $V_{i}=r_{i}\times r_{o}\times L=0.8\;\mathrm{mm}\times 1.6\;\mathrm{mm}\times 1000\;\mathrm{mm}$ (platinum cross-linked silicone, Versilic SPX-50, Saint Gobain Performance Plastics),
where $V_{i}$ is the inner volume, $r_{i},\;r_{o}$ are the inner and outer radii, and L is the length of the membrane tube.

Gas-tight polyurethane tubes ($r_{i}=1\;\mathrm{mm}$, Festo, Esslingen, Germany) were used to connect the membrane tubes with pinch valves. These valves (108P8NO12- 01B, Bio-Chem Fluidics, Inc., Boonton, NJ, USA) were used to enable the purging of the membrane tubes with dried compressor air that escapes downstream from the purge gas outlet into the air. The pressure sensors were connected with C-flex tubing ($r_{i}=0.4\;\mathrm{mm}$, Saint Gobain Performance Plastics). The inner volumes of the tubing, fittings, and pressure sensors added a dead volume of about $V_{d}=1900$ mm^3^ to the inner volume of each chamber.

Pressure sensors of type AMS 5812-0000-D-B (range Δp ±5.17 hPa, precision ±2% of full scale, Amsys GmbH & Co, Mainz, Germany) were used to measure the pressure difference $\Delta p_{\mathrm{TA}}$ between the chambers in the test and air containers and the pressure difference $\Delta p_{\mathrm{VA}}$ between the vapor and air containers.

Dried air from a compressor (RH≈4%) was used as the purge gas for all chambers. The airflow was adjusted using a mass flow controller (MFC 8710, range 0–5 l/min for air, Bürkert Fluid Control Systems, Ingelfingen, Germany) and controlled by a glass tube dipped into an open water-filled bottle. In this way, a pressure buildup of 20 hPa upstream from the cells was created.

Each cyclic measurement consisted of 110 s for purging the chambers, about 6 s for the consecutive pressure measurement, and a pause of about 4 s, resulting in a total sampling time of 120 s. The initialization of the measurement step caused pressure equilibration within the chambers after the pinch valves were closed. To minimize the influence of this relaxation process on the measurement signal, an offset time of 0.1 s was applied between the closing of the pinch valves and the start of pressure registration. To control the cyclic measurement (i.e., to switch the pinch valves and register the pressure differences $\Delta p_{\mathrm{TA}}\left(t\right)$ and $\Delta p_{\mathrm{VA}}\left(t\right)$), the actuator unit described in [25] was used. The differential pressure changes $\propto_{\mathrm{TA}}$ and $\propto_{\mathrm{VA}}$ were calculated from the measured pressure–time curves $\Delta p_{\mathrm{TA}}\left(t\right)$ and $\Delta p_{\mathrm{VA}}\left(t\right)$ (for more detailed information, see [25]), where a correction of these pressure changes to the inner volume $V_{i}$ of the membrane tubes was carried out by multiplication with ($V_{d}+V_{i})/V_{i}$, and stored on a laptop.

Sensors for temperature and relative humidity (EE060, E+E Elektronik, Engerwitzdorf, Austria) were placed in the upper part of each container. The measurement range for humidity ranged from zero to full saturation, with a precision of ±2.5% of the measured value. The temperature range was between −40 and 60 °C, with a precision of ±0.3 K. The outer air pressure was measured using an HCA-BARO series air pressure sensor (range 600–1100 hPa, accuracy ±1% full-scale span, First Sensor, Berlin, Germany). The measurements were recorded on a PC using DASYLab 10.0 (dasylab.com).

### 2.3. Theory

To investigate the response of the MHS, depending on the aggregate state of water, the relationship between the pressure change α measured within the membrane tube and the steady-state flow Q [mol/s] of the gas components of air through its tubular wall must be considered. A pressure change α develops in the measurement step, close to the steady-state gas flow, and depends on the change in the mole number dn/dt=Q of gas in the inner void of the membrane tube $V_{i}$. With respect to the initial gas pressure $p_{i}$ [hPa] in the chamber and the molar volume $V_{m}$ [L/mol], the relationship between the change in the mole number and the change in pressure follows according to the ideal gas law as:

(2) $$\frac{dn}{dt}=Q=\frac{1}{V_{m}}\frac{V_{i}}{p_{i}}\alpha$$

Based on the assumptions of (i) the adsorption–desorption equilibria for the gas components in the air and the surfaces of the membrane, (ii) the applicability of the solution-diffusion model for gas transport through a non-porous membrane, and (iii) the validity of the superposition principle, the steady-state diffusive flow Q through the wall of the membrane can be expressed as the weighted sum [37] of the differences in the outer $p_{k}^{o}$ and inner $p_{k}^{i}$ partial pressures [hPa] of the ambient gas components (“k”):

(3) $$Q=\frac{1}{RT}\frac{2\pi \cdot L}{\ln{\;r_{o}/r}_{i}}\sum_{k}P_{k}\;(p_{k}^{o}-p_{k}^{i}),$$

where $P_{k}$ are the permeabilities (given in [m^2^/s] in Table 1 of [25]), R is the universal gas constant, and T [K] is the absolute temperature. The partial pressures $p_{k}^{o}=\chi_{k}^{o}\cdot p_{air}$ depend on the mole fractions $\chi_{k}^{o}$ of the components of the air and its pressure $p_{air}$ in the surroundings of the membrane tube. The partial pressures $p_{k}^{i}=\chi_{k}^{i}\cdot p_{i}$ in the purged membrane tube depend on the mole fractions $\chi_{k}^{i}$ of the purge gas and its mean pressure $p_{i}$. To purge a gas through the chamber, its inlet pressure $p_{in}$ must be higher than the outlet pressure $p_{out}$. Assuming a linear pressure drop along the tubular membrane and neglecting the pressure drops in the valves, fittings, and connecting tubes of the chamber, then for the air as purge gas escaping into the surroundings ($p_{out}=p_{air}$), the mean pressure in the membrane tube is equal to $p_{i}=\left(p_{in}+p_{air}\right)/2$.

***Simplification:*** With respect to the permeability of water $P_{w}$ and the ratios $f_{kw}=P_{k}/P_{w}$, Equation (3) can be split into two terms representing the proportions of water $\left(W\right)$ and the components of dry air $\left(G\right)$ in the flow Q:

(4) $$Q=\frac{1}{RT}\frac{2\pi L\;P_{w}}{\ln r_{o}/r_{i}}\{\underset{\begin{array}{c} |\\ =W \end{array}}{\underset{⎵}{\left(p_{w}^{o}-p_{w}^{i}\right)}}+\underset{\begin{array}{c} |\\ =G \end{array}}{\underset{⎵}{\sum_{k\neq w}f_{kw}(p_{k}^{o}-p_{k}^{i})}}\}.$$

The ambient air around the membrane tube contains vapor at the saturation vapor pressure $p_{w}^{o}=e_{w}^{\prime }$, according to Equation (1). For air as a purge gas at the same temperature and relative humidity $RH_{i}$, the vapor pressure in the chamber is $p_{w}^{i}=e_{w}^{\prime }\cdot a_{w}^{i}$, where $a_{w}^{i}=RH_{i}/100\%$ is the water activity in this air. The gas pressures on both sides of the membrane are determined by the air pressure, which is assumed to remain constant during measurement. Then, the positive difference in the water mole fractions between the two sides, $\Delta \chi_{w}\approx e_{w}^{\prime }\left(1-a_{w}^{i}\right)/p_{air}$, describes the additional dilution of the gas components in the outer air by a proportion $\left(1-\Delta \chi_{w}\right)$. In terms of the mole fractions $\chi_{k}^{i}$ of air in the chamber, the composition of the outer air is then $\chi_{k}^{o}=\delta_{kw}\Delta \chi_{w}+\left(1-\Delta \chi_{w}\right)\chi_{k}^{i}$, where $\delta_{kw}$ is the Kronecker delta. Replacing the partial pressures in Equation (4) by the respective mole fractions enables the comparison of the contributions of the terms W and G:

(5) $$\frac{G}{W}=\left(\frac{1}{e_{w}^{\prime }}\frac{p_{air}-p_{i}}{1-a_{w}^{i}}-1\right)\;\;\sum_{k\neq w}f_{kw}\;\chi_{k}.$$

According to Table 1 in [25], the ratio $f_{N2w}=P_{N2}/P_{w}$ is 0.008 and $f_{O2w}=P_{O2}/P_{w}$ is 0.017. In terms of the mole fractions of the main components of dry air (N_2_, O_2_), the weighted sum in Equation (5) is about $0.8\cdot f_{N2w}+0.2\cdot f_{O2w}\approx 0.01$. With a purge gas overpressure $p_{in}-p_{air}$ of 20 hPa at the inlet of the chamber (see Section 2.2), a vapor pressure $e_{w}^{\prime }=31.7$ hPa at a temperature of 25 °C, an air pressure of 1013 hPa, and an $RH_{i}$ of 10% in the purge gas, the value of the ratio in Equation (5) is $\left|G/W\right|<0.017$. The term G can therefore be neglected, and the flow through the membrane can essentially be attributed to the difference in the partial pressures of the vapor:

(6) $$Q=\frac{1}{RT}\frac{2\pi L\;}{\frac{1}{P_{w}}\ln\frac{r_{o}}{r_{i}}}\;(p_{w}^{o}-p_{w}^{i}).$$

***Case 1: Diffusive depletion zone***. A diffusive depletion zone of vapor can develop around the membrane tube in stagnant air as a result of the diffusive vapor transport towards its membrane surface, which acts as a sink due to the diffusive vapor flow through it. This zone can be described as a film with an outer radius $r_{f}$ [m] around the membrane tube at which the vapor pressure approximates the saturation vapor pressure $e_{w}^{\prime }$. If $D_{f}$ [m^2^/s] is the diffusion coefficient of vapor for the air in this zone, the radial flow $Q_{f}$ can be expressed in an analogous way to Equation (6), as follows:

(7) $$Q_{f}=\frac{1}{RT}\frac{2\pi L}{\frac{1}{D_{f}}\ln\frac{r_{f}}{r_{o}}}\;(e_{w}^{\prime }-p_{w}^{o}).$$

In the steady state, the flow through the film is the same as that through the membrane, and hence:

(8) $$Q=\;\frac{1}{RT}\frac{2\pi L}{\frac{1}{D_{f}}\ln\frac{r_{f}}{r_{o}}+\frac{1}{P_{w}}\ln\frac{r_{o}}{r_{i}}}(e_{w}^{\prime }-p_{w}^{i})=Q_{f}.$$

Combining Equations (2) and (6) gives the pressure change within a cell regardless of any additional outer film layer. This condition was adjusted experimentally in the test container, as shown in Figure 1, where the membrane tube was surrounded by a liquid (“liq”) water layer. The pressure change ∝ can therefore be denoted as $\propto_{liq}$, and the experimental conditions in this container are considered to depend on the vapor pressure $e_{w,T}^{\prime }$ and the temperature $T_{T}$. It follows that:

(9) $$\alpha_{liq}=\frac{T_{0}}{p_{0}}\frac{p_{i}}{T_{T}}\frac{e_{w,T}^{\prime }}{\tau }(1-a_{w}^{i}),$$

where ($T_{0},\;p_{0})$ considers the standard conditions (273.15 K, 1013.25 hPa) and *τ* [s]:

(10) $$\tau =\;\frac{V_{i}\cdot \ln r_{o}/r_{i}}{2\pi L\cdot P_{w}},$$

is a time parameter that characterizes the response of the cell. The water permeability of the membrane can be obtained for this case from the measurement in the test container and can be estimated using Equations (9) and (10).

Combining Equations (2) and (8) and considering the experimental conditions (vapor pressure $e_{w,V}^{\prime }$ and temperature $T_{V}$) for the vapor container shown in Figure 1 gives a pressure change $\propto_{gas}$, measured with a membrane tube that is surrounded by air (“gas”) and situated in a stagnant gaseous film forming the diffusive depletion zone:

(11) $$\alpha_{gas}=\frac{T_{0}}{p_{0}}\frac{p_{i}}{T_{V}}\frac{e_{w,V}^{\prime }}{\tau }\frac{\ln\frac{r_{o}}{r_{i}}}{\frac{P_{w}}{D_{f}}\ln\frac{r_{f}}{r_{o}}+\ln\frac{r_{o}}{r_{i}}}(1-a_{w}^{i}).$$

The pressure changes in Equations (9) and (11) can be expressed independently of the varying outer conditions as:

(12) $$\alpha_{liq}^{*}=\frac{p_{0}}{T_{0}}\frac{T_{T}}{p_{i}\;e_{w,T}^{\prime }}\alpha_{liq},\;\alpha_{gas}^{*}=\frac{p_{0}}{T_{0}}\frac{T_{V}}{p_{i}\;e_{w,V}^{\prime }}\alpha_{gas}.$$

The flow $Q_{f}$ through the film in Equation (7) is equal to that given by Equation (8). Thus, for the cell installed in the vapor container holds:

(13) $$\frac{D_{f}}{P_{w}}\frac{\ln r_{o}/r_{i}}{\ln r_{f}/r_{o}}=\frac{p_{w,V}^{o}-p_{w}^{i}}{e_{w,V}^{\prime }-p_{w,V}^{o}}.$$

The same left-side term results in

(14) $$\ldots =\frac{\alpha_{gas}^{*}}{\alpha_{liq}^{*}-\alpha_{gas}^{*}},$$

when comparing the pressure changes in Equations (9), (11) and (12). Hence, Equations (13) and (14) enable the estimation of the water activity $a_{w}^{s}$ at the outer surface (“*s*”) of the membrane tube in the vapor container, as follows:

(15) $$a_{w}^{s}=\frac{\alpha_{gas}^{*}}{\alpha_{liq}^{*}}(1-a_{w}^{i})+a_{w}^{i}.$$

From Equation (14), the film thickness $\delta_{f}=r_{f}-r_{o}$ can be determined as:

(16) $$\delta_{f}=r_{o}\left(e^{\gamma }-1\right),\;\gamma =\frac{D_{f}}{P_{w}}\left(\frac{\alpha_{liq}^{*}}{\alpha_{gas}^{*}}-1\right)\cdot \ln\frac{r_{o}}{r_{i}}.$$

***Case 2: Preferred absorption of water from a liquid.*** An alternative to *Case 1* is that the sorption behavior of the hydrophobic membrane material may change in the case of direct contact with liquid water with respect to its sorption behavior for the single water molecules of the gaseous surroundings that touch the membrane surface. If the stagnant gaseous film can be neglected around the membrane tube in the vapor container, Equations (11) and (12) give

(17) $$\alpha_{gas}^{*}=(1-a_{w}^{i})\cdot \tau^{-1},$$

and the permeability in Equation (10) is determined by the pressure change in the vapor container. 

The changed absorption efficiency (i.e., the changed density of the water molecules in the membrane surface of a liquid water-environment with respect to a saturated vapor environment) can be expressed in an analogous way to the water activity $a_{w}^{s}$ at the outer membrane surface (*Case 1*) by an equivalent parameter $a_{w}^{b}$ representing the water activity of the boundary layer (“*b*”) of the membrane in the test container:

(18) $$\alpha_{liq}^{*}=(a_{w}^{b}-a_{w}^{i})\cdot \tau^{-1}.$$

A comparison of Equations (17) and (18) enables the calculation of this equivalent based on simultaneous measurements in the test and vapor containers:

(19) $$a_{w}^{b}=\frac{\alpha_{liq}^{*}}{\alpha_{gas}^{*}}(1-a_{w}^{i})+a_{w}^{i}.$$

If $a_{w}^{b}\neq 1$, the density of the water molecules between the chains of polymer in the membrane surface can then be described in terms of the equivalent of an over- or under-saturated vapor.

As shown in Figure 1, the evolutions of the pressure differences $\Delta p_{\mathrm{TA}}\left(t\right)$ and $\Delta p_{\mathrm{VA}}\left(t\right)$ between the various containers were measured. These measurements indicate a transition from a simple pressure change $\alpha_{T/V}=dp_{T/V}/dt$ within a single chamber to a differential pressure change $\alpha_{\mathrm{TA}/\mathrm{VA}}=d(\Delta p_{\mathrm{TA}/\mathrm{VA}})/dt$ between the respective chamber pairs. Transforming $1-a_{w}^{i}$ in Equations (9) and (11) for this measurement arrangement gives $1-a_{w}^{i}+\varphi_{T/V}\left(a_{w}^{i}-a_{w,A}\right)$, with water activity $a_{w,A}$ in the air container, and the ratio

(20) $$\varphi_{T/V}=\frac{T_{T/V}}{T_{A}}\frac{e_{w,A}^{\prime }}{e_{w,T/V}^{\prime }}$$

of the conditions, under which the water permeation takes place inside the respective container pairs. For the same container temperatures, these ratios approximate $\varphi_{T/V}\to 1$. Then, the (possibly changing) vapor pressure of the purge gas no longer influences the measurement value; instead, it is determined by the RH in the air container, which then acts as a common reference point for the cells in the test and vapor containers. In this case, the measured pressure changes depend on the vapor pressure differences between the respective container pairs (T, A) or (V, A):

(21) $$\Delta e_{w,\mathrm{TA}/\mathrm{VA}}^{\prime }=e_{w,T/V}^{\prime }(1-a_{w,A}^{}).$$

## 3. Experimental Investigation

### 3.1. Experiments

Each experiment consisted of two phases. In Phase 1, evaporation from the liquid water layers was used to create vapor-saturated conditions in the test and vapor containers. In Phase 2, the water level in the test container was elevated above the membrane tube. The relative humidity $RH_{A}$ around the membrane tube in the air container was adjusted with the membrane dryer. To create the change from Phase 1 to Phase 2, the covering (consisting of cloths) was removed from the container stack. A peristaltic pump was connected to fill up the test container with water. The filling process was observed visually and was stopped when the membrane tube was covered with water. The peristaltic pump was then removed, and the container stack was covered again with cloths.

**Figure 2:**
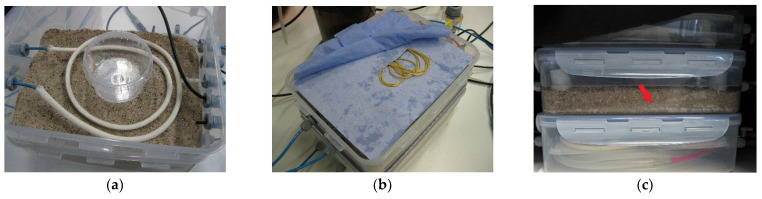
Test container used for Experiment 3. (**a**) A membrane tube integrated into a cotton hose was placed onto a sand layer. The plastic vessel in the center served as a water storage. (**b**) After covering the membrane tube with sand and installing the EE060 sensor above it, water was added to the plastic vessel and connected by a capillary thread (yellow), with a cloth placed on a grid to allow the water to evaporate into the closed test container for Phase 1. (**c**) The light areas (marked with a red arrow) within the watered sand pack in Phase 2 indicate residual amounts of trapped air.

Three experiments were performed. In Experiment 1, membranes of the Set 1 type were used. Water was filled to a height of 1 cm in both the test and vapor containers, resulting in equilibration of the vapor-saturated air in both containers. Pressure change measurements were performed in the gaseous environments around the membrane tubes in both containers in Phase 1. Water was then added to the test container for about 10 min, until the membrane tube was covered with liquid water, and the pressure response for Phase 2 was observed in this container. As a simultaneous reference for the vapor-saturated air, the response of the cell in the vapor container was recorded. In Experiment 2, membranes of the Set 2 type were used in the same manner, to allow us to observe the pressure changes for another membrane with a different geometry and fabrication. In a near-application scenario, the response of the Set 1 membranes was tested in Experiment 3 for a porous substrate. In this test scenario, the membrane tubes were covered with loosely fitting cotton hoses. Then, the membrane tube in the test container was placed in dry sand. Figure 2a shows a membrane tube above a 1 cm sand layer in the test container. The vessel in the center of the container allowed the storage of water for humidification while avoiding contact with the surrounding sand. Using a thread (shown in yellow in Figure 2b), this water was raised by capillary action above the body of the sand and spread over the area of the cloth (shown in blue), which was placed on a plastic grid. From there, the water evaporated. In Phase 1, the observation was started after vapor saturation was achieved. In Phase 2, water was slowly added to the test container from the bottom to the top, over a period of about 30 min. Filling was stopped when the water level exceeded the surface of the sand body. Figure 2c shows the wet sand. The light areas indicate trapped residual air that could not be prevented during water filling.

### 3.2. Data Processing

***Data selection:*** Within the range of validity of the simplified theory, the pressure change is dependent on the difference in the vapor pressure of water $\Delta e_{w}^{\prime }$, which in turn depends mainly on the respective container temperatures. External disturbances to the experiment (e.g., failure to achieve vapor saturation in the test or vapor containers) therefore result in differences between the expected and observed pressure changes. To prepare a dataset for further consideration, a non-disturbed region of data had to be selected. To achieve this, arbitrary selected ranges of data were approximated separately for each experimental phase and container (V, T) with respect to the differential vapor pressures $\Delta e_{w,\mathrm{TA}}^{\prime }$ and $\Delta e_{w,\mathrm{VA}}^{\prime }$ (according to Equation (21)) by a linear fit model:

(22) $$\alpha (\Delta e_{w}^{\prime })=(a_{0}\pm \delta a_{0})+(a_{1}\pm \delta a_{1})\;\Delta e_{w}^{\prime }.$$

The respective vapor pressures were calculated based on the Magnus formula in Equation (1), using the individual container temperatures and relative humidities (recorded independently with the EE060 sensors) and the air pressure. The measured pressure changes at the beginning and end of each experiment were excluded from further consideration if they differed significantly (systematically) from the calculated pressure changes.

***Correction to the experimental conditions:*** The measured and calculated data were corrected using Equation (12) with regard to the experimental conditions: the relative humidity in the air container, the temperatures in the containers, the air pressure and the purge gas pressure. To improve the comparability of the different membrane sets, the pressure changes were multiplied by the time parameter τ for the respective cell according to Equation (10). 

***Adjustment of responses:*** Systematic differences between the pressure changes $\propto_{\mathrm{TA}}$ and $\propto_{\mathrm{VA}}$ may arise due to geometric differences between the cells and the purge gas flows, the different detection behaviors of the pressure sensors, etc. As the same external conditions are used in Phase 1, the modified pressure responses shown in Equation (12), $\alpha_{\mathrm{TA}}^{*}$ and $\alpha_{\mathrm{VA}}^{*}$, must be the same in this phase. Taking into account that (i) any pressure change must disappear when RH is the same on both sides of the membrane, and (ii) the linearity of the measurement signal with RH [25], adjusting the membrane set requires the equivalence of the averaged pressure changes ${\bar{\alpha }}_{\mathrm{TA}}^{*}$ and ${\bar{\alpha }}_{\mathrm{VA}}^{*}$. This makes it possible to determine a scaling factor κ for Phase 1, where $\kappa \cdot {\bar{\alpha }}_{\mathrm{TA}}^{*}={\bar{\alpha }}_{\mathrm{VA}}^{*}$, and thus to adjust the pressure change measured between the various cells as follows:

(23) $${\hat{\alpha }}_{\mathrm{TA}}^{*}=\kappa \cdot \alpha_{\mathrm{TA}}^{*}.$$

## 4. Results

### 4.1. Experiments

#### 4.1.1. Experiment 1

**Figure 3:**
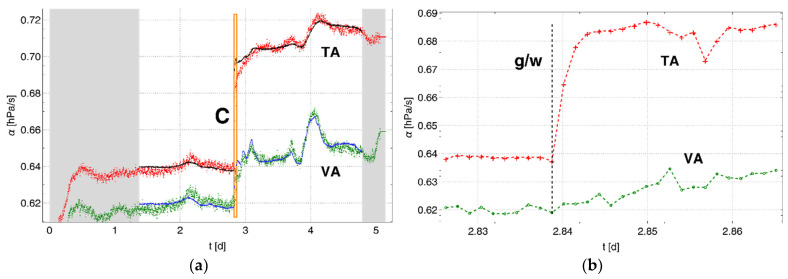
(**a**) Pressure changes analyzed in the test container (TA, red) and the vapor container (VA, green) versus the air container. Gray regions represent data that were not included in the investigation. Solid lines (TA: black, VA: blue) represent the calculated pressure changes. The data within the orange box marked C and shown in detail in (**b**) demonstrate the step-like increase in the pressure change (red) when water was added to cover the surface of the membrane tube in the test container (black dashed line, marked g/w). However, the pressure change (green) in the vapor container changed gradually.

Figure 3a shows the measured pressure changes $\propto_{\mathrm{TA}}$ (red) and $\propto_{\mathrm{VA}}$ (green). The data within the orange box, which are shown in more detail in Figure 3b, were measured during the period in which water was added to the test container (event “C”). This event C divides the data associated with Phases 1 and 2 of the experiment. The dashed black line marked “g/w” in Figure 3b indicates the time at which the water filling of the test container must have caused the replacement of the vapor-saturated air around the membrane tube with water, which was followed by a rapid increase in the pressure change. The subset of the data used for further consideration was selected from the region ranging from 1.4 to 4.8 d. The ranges marked in gray in Figure 3a represent the data that are outside of this region. The means and standard deviations of the subsets of data for both phases are given in Table A1 in Appendix A. The suitability of the data in this subset was tested based on the fit of $\alpha (\Delta e_{w}^{\prime })$, using Equation (22). It can be seen from Figure 3 that there is a good match between the experimental and calculated data (TA: black line, VA: blue line) based on the linear fits of $\alpha (\Delta e_{w}^{\prime })$. The fit parameters are shown in Table A2 in Appendix A. The low correlation coefficients for the fits for Phase 1 in Table A2 result from the comparatively small changes of vapor pressure in this phase with respect to Phase 2.

**Figure 4:**
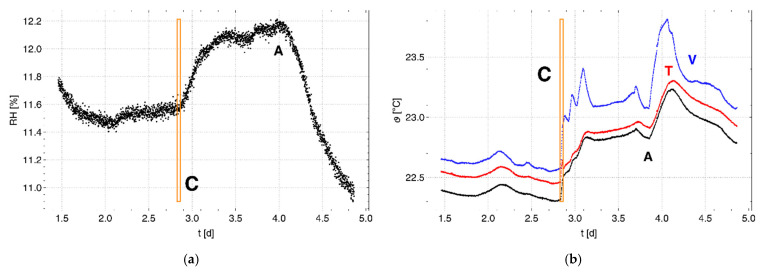
(**a**) Relative humidity in the air container and (**b**) temperatures in the containers of the test system (T: red, V: blue, A: black). The orange box marked C indicates the change from Phase 1 to Phase 2.

Figure 4a shows $RH_{A}$ for the air container. The values for RH in the vapor and test containers were constant at 100%. Figure 4b shows the temperatures in the containers (A: black, T: red, V: blue). These temperatures vary during Phase 1 within the precision of the sensor. A disturbance in the temperature during the manipulation of the container stack due to water filling is clearly visible. Compared to Phase 1, the temperature in the vapor container follows the room temperature with more dynamics during Phase 2 than that in the subjacent containers (T, A) of the container stack. This can be attributed to the change in the thermal insulation after filling with water and the increased thermal inertia of the test container, which shielded the air container from above. A comparison with Figure 3 confirms that the pressure changes follow the evolution of the temperature in the containers, which controls the vapor pressure.

Based on the respective mean values of the container temperatures and the air pressure in Table A1, the ratios were estimated using Equation (20) for Phase 2 for the test container ($\varphi_{T}=0.99$) and the vapor container ($\varphi_{V}=0.98$), with respect to the air container. Both ratios show similar conditions for water permeation in the different containers, but the larger temperature difference between the vapor and air containers led to a somewhat lower value of the ratio $\varphi_{V}$. Nevertheless, the ratios show that to a good approximation, the experimental setup allows the use of the relative humidity in the air container rather than that of the purge gas as a reference for measurement, meaning that the applicability of Equation (21) holds. 

**Figure 5:**
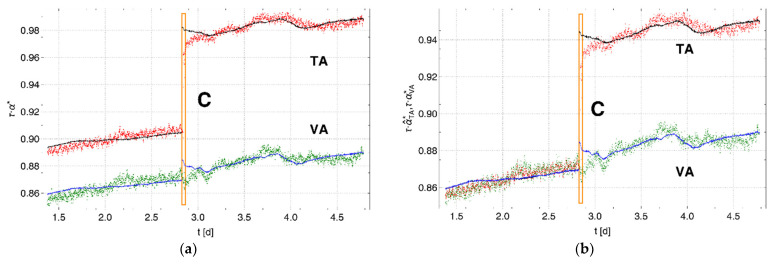
Pressure changes $\alpha_{\mathrm{TA}}^{*}$ (red) and $\alpha_{\mathrm{VA}}^{*}$ (green) multiplied by the time parameter τ of the Set 1 cell: (**a**) corrected for environmental influences and (**b**) adjusted to each other. The solid lines (TA: black, VA: blue) represent the respective calculated data. The data region within the orange box marked C represents the transition from Phase 1 to Phase 2, where the vapor-saturated air in the surroundings of the membrane tube in the test container was replaced by liquid water.

The measured and calculated pressure changes were corrected for the varying experimental conditions using Equation (12) and, for better comparability between the different cells, were multiplied by the time parameter τ=44.9 s, according to Equation (10), for the vapor-saturated air in the surroundings of the membrane tube. Figure 5a shows the dimensionless data $\tau \cdot \alpha^{*}$. The variations in the primary data shown in Figure 3 were strongly reduced. In addition, the jump in the pressure change $\propto_{\mathrm{VA}}$ in the vapor container at point C has disappeared in Figure 5, meaning that this jump can be attributed to the change in temperature in the containers during/after filling with water. Figure 5b shows the adjusted pressure change ${\hat{\alpha }}_{\mathrm{TA}}^{*}$ with respect to $\alpha_{\mathrm{VA}}^{*}$ in dimensionless notation. The scaling factor according to Equation (23) was κ=0.9614.

#### 4.1.2. Experiment 2

To prove the existence of a phase-dependent discontinuity, a similar experiment was carried out with cells prepared using the different Set 2 membranes. The experiment was run using the same container setup. The measurement and the data analysis were carried out in the same way as for Experiment 1. The results shown in Appendix B confirm the results of Experiment 1 for an expanded temperature range.

#### 4.1.3. Experiment 3

If one neglects their points of contact on the support grids, the membrane tubes of MHS in Experiments 1 and 2 can be considered to be surrounded by the gaseous or the aqueous phase. Experiment 3 was therefore performed to analyze the response under the conditions that could be expected if the membrane tubes were placed within a moist substrate (e.g., litter or soil). The experimental results are shown in Appendix C.

**Figure 6:**
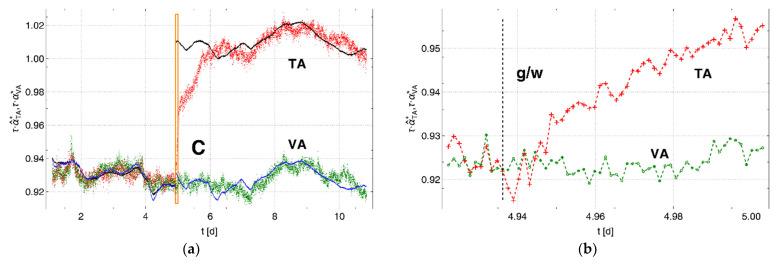
(**a**) Pressure changes ${\hat{\alpha }}_{\mathrm{TA}}^{*}$ (red) and $\alpha_{\mathrm{VA}}^{*}$ (green) adjusted to each other and multiplied by the time parameter τ for Set 1 for both phases of the experiment. The solid lines (TA: black, VA: blue) represent the calculated data. The data region within the orange box marked C indicates the transition between Phases 1 and 2, where the vapor-saturated air in the surroundings of the membrane tube in the test container was replaced by liquid water. The region of data within the orange box is shown in more detail in (**b**). The black line marked g/w indicates the time at which liquid water starts to cover the membrane surface of the membrane tube.

To adjust the pressure changes, a scaling factor of κ=1.075 was determined for this experiment. A comparison between this scaling factor and that of Experiment 1 (κ= 0.9614) may indicate a slight influence from the sand pack that surrounds the membrane tube in the test container. However, after adjustment of the pressure changes, the temperature-controlled behavior of the pressure change is almost identical for the air-surrounded (Phase 1) membrane tubes, as shown to the left of Figure 6a. In a similar way to Experiments 1 and 2, the adjustment makes the phase-dependent jump visible, although in this case it is apparent that the change in response occurs more slowly over time, unlike the calculated, temperature-dependent response, as shown in Figure 6.

### 4.2. Phase-Dependent Behavior of the PDMS Membrane

#### 4.2.1. Analysis of Boundary Layer Behavior

The membrane tube-based chambers are flushed with air, which causes the exchange of molecules near the inner membrane surfaces. In contrast, their outer membrane surfaces are surrounded by stagnant air or water. If there is a diffusive depletion zone near the membrane surfaces, it will be formed most strongly close to the outer membrane surface, which is surrounded by stagnant air (i.e., within the vapor container). No such depletion zone can be formed around the membrane tube in the test container, which is surrounded by water. The latter cell can therefore serve as a reference in this context. To examine the existence of the diffusive depletion zone (*Case 1*), the permeability of the Set 1 membranes was estimated using Equation (10) as $P_{w}=1.59\cdot 10^{-4}$ cm^2^/s for the experimental data of the test container (Table A1). This calculated value is of the same order of magnitude as the vapor permeability given in the literature (e.g., in [31]). The coefficient of diffusivity of water molecules in air is 0.2178 cm^2^/s ($p_{0}=$ 1013.25 hPa, $T_{0}=$ 273.15 K), according to Table 8 in [38], and the author recommended a conversion rule of $D\left(p,T\right)=D\left(p_{0},T_{0}\right)\left(p_{0}/p\right){\left(T/T_{0}\right)}^{1.81}$ with respect to the actual p-T conditions. This conversion gives a value of $D_{f}=0.256$ cm^2^/s, for a mean temperature of 23.3 °C in the vapor container and an air pressure of 999 hPa (Table A1, Phase 2). The ratio $\alpha_{gas}^{*}/\alpha_{liq}^{*}$ in Equation (15), which is given by $\alpha_{\mathrm{VA}}^{*}/{\hat{\alpha }}_{\mathrm{TA}}^{*}$ of the adjusted membrane set, was determined from the experiment as 0.899. According to Equation (15), this ratio will give a value for the water activity of $a_{w}^{s}=0.91$ near the surface of the membrane in the vapor container. From Equation (16), one finds that a film thickness of $\delta_{f}=2\times 10^{29}$ m will result. Thus, the concentration gradient of vapor in the film is negligible, and the outer vapor concentration must be considered as not being influenced by the permeation process through the membrane, meaning that it is independent of the location in the container. The reference measurements (Table A1) show an *RH* of 100% rather than a value of $100\%\times a_{w}^{s}=91\%$ close to the outer membrane surface. This indicates that the drop in the measured pressure changes cannot be explained by the presence of a diffusive depletion zone around the membrane. This result also implies that no such diffusive depletion zone will be formed in the membrane tubes, i.e., near the inner membrane surfaces.

If the existence of a depletion zone can be neglected, however, the water permeability of the membrane is given by the measurement in the vapor container. Using Equation (11) for $r_{f}\to r_{0}$ yields a value of $P_{w}=1.43\cdot 10^{-4}$ cm^2^/s. In addition, a preferred absorption of water from a liquid environment (*Case 2*) must then explain the phenomenon. The investigation gives an equivalent $a_{w}^{b}$ of the water activity for the membrane surface of 1.1, according to Equation (19). Thus, the experiment indicates an apparent oversaturation of vapor of 10% if the membrane is surrounded by liquid water.

#### 4.2.2. Differentiation of Saturated Vapor from Liquid Water

The experiments demonstrate that the cause of the change in vapor permeation must be attributed to mechanisms at the membrane surface, rather than to the presence of a depletion zone outside the membrane. Membrane tubes of this type can therefore be used to measure the physical state of water. To achieve this, two such membrane tubes must be exposed to a volume containing water in the unknown physical state and water in a known physical state as an internal reference. The MHS already benefits from the use of such an internal reference, as it makes measurements of RH independent of temperature.

**Figure 7:**
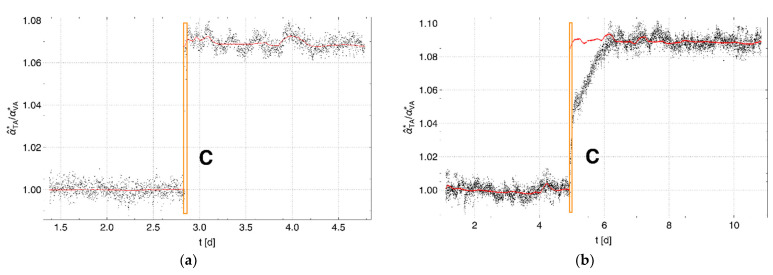
Ratio ${\hat{\alpha }}_{\mathrm{TA}}^{*}/\alpha_{\mathrm{VA}}^{*}$ (black) for the adjusted cells (**a**) for Experiment 1 and (**b**) for Experiment 3. Solid lines (red) represent the respective ratio from the calculated data. The region of data within the orange box marked C indicates the change of the saturated vapor in the test container to a liquid water environment in the surroundings of the membrane tube, while the tube in the vapor container is always surrounded by vapor-saturated air. Unlike in Experiment 1, the membrane tube within the test container was placed in a body of sand in Experiment 3 and surrounded by cotton fabric. While the calculated results (red) for both experiments show a sudden jump at point C, the measurements followed the calculated values only in Experiment 1. The significant deviation in Experiment 3 must be due to (vapor-saturated) air partly covering the membrane surface after watering at C.

Figure 7a shows the ratio ${\hat{\alpha }}_{\mathrm{TA}}^{*}/\alpha_{\mathrm{VA}}^{*}$ of the pressure changes (shown in black) for the adjusted set of cells used in Experiment 1, and Figure 7b shows the ratio for Experiment 3. The red lines show the respective ratios from the associated calculated pressure changes. The measured and calculated data correspond to a gaseous environment for Phase 1 and a liquid environment for Phase 2 around the membrane tube in the test container, whereas the environment in the vapor container was always gaseous. The measured and calculated data show a perfect match for both phases of Experiment 1. A significant jump of about 7% occurred at the change from vapor to liquid water in the membrane surroundings.

In contrast, Experiment 3 in Figure 7b shows a significant difference between the ratios calculated based on the state of aggregation of the water and those determined by measurement after watering the sand in the test container. It can be seen from Figure 2c that the watering of the sand body led to the trapping of the residual distributed air. The enclosure of the membrane tube with a fine-mesh hydrophilic hose obviously also allowed air to be trapped near the membrane wall. This air formed a gaseous environment for part of the membrane surface. Due to the gradual detachment of the air, this portion reduced over time and disappeared completely after about one day, i.e., the environment of the membrane then contained only liquid water.

Taking into account the two known limiting situations (i.e., only vapor-saturated air and only liquid water), which correspond to the mean ratios $\gamma_{g}=\mu \left({\hat{\alpha }}_{\mathrm{TA}}^{*}/\alpha_{\mathrm{VA}}^{*}\right)$ for Phase 1 and $\gamma_{w}=\mu ({\hat{\alpha }}_{\mathrm{TA}}^{*}/\alpha_{\mathrm{VA}}^{*}$) for Phase 2 after complete saturation (>day 6), respectively, an average saturation of liquid water $s_{w}$ near the membrane surface can be derived from the ratio $\gamma ={\hat{\alpha }}_{\mathrm{TA}}^{*}\left(t\right)/\alpha_{\mathrm{VA}}^{*}\left(t\right)$, as follows:

(24) $$s_{w}=\frac{\gamma -\gamma_{g}}{\gamma_{w}-\gamma_{g}}$$

**Figure 8:**
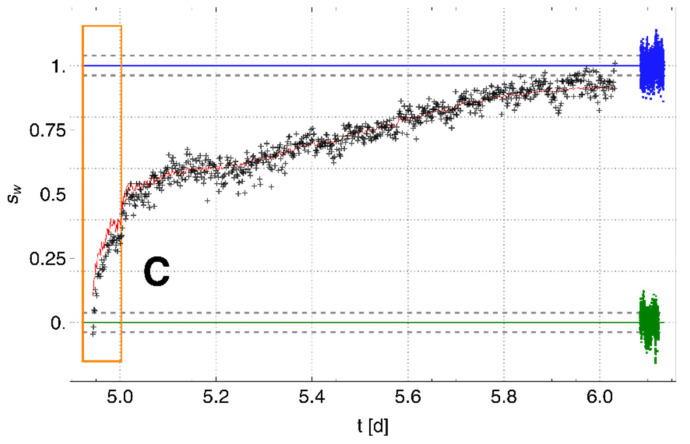
Increasing water saturation (black) around the membrane tube in the test container for Experiment 3. For comparison, the respective signal fluctuations for complete vapor saturation (green) and complete water saturation (blue) around the membrane are shown compressed in time on the right. Standard deviations are shown as dashed lines. The red line shows the mean evolution of water saturation. After a fast increase in saturation during watering (orange box, marked C), the slow further increase can be attributed to the comparatively slow displacement of the remaining residual air around the membrane surface by water.

The black crosses in Figure 8 show the saturation around the membrane (calculated using Equation (24)) in the test container for the time during which the ratio ${\hat{\alpha }}_{\mathrm{TA}}^{*}/\alpha_{\mathrm{VA}}^{*}$ changed (day 5 to day 6). During watering, the displacement of air by the rising water table resulted in a water saturation of about 50% in the surroundings of the membrane before further degassing was stopped by the entrapment of the remaining air. After this point, further saturation took place slowly in the now stagnant pore-water/air environment of the membrane. For comparison, the fluctuation in the calculated saturation is shown to the right, compressed in time, for complete vapor saturation (green) and water saturation (blue). Based on a rough estimate of the average evolution of the saturation (red line, estimated with an arbitrarily adjusted edge-preserving bilateral filter [39]), the standard deviation can be estimated for the calculated water saturation as 0.035. This is in alignment with the standard deviations of 0.038 and 0.039 for complete gas ($s_{w}=0$) and complete water saturation ($s_{w}=1$), respectively.

## 5. Discussion

The present study identifies a previously unknown phenomenon. The conceptual framework of investigations and the experimental design resulted in the following uncertainties and limitations: (i) most of the uncertainty in the measured data seems to be caused by the temperature differences between the containers of the experimental setup. (ii) The difference in the responses in Experiments 1 and 3 indicates a possible influence from the substrate surrounding the membrane tubes. (iii) Due to the exponential dependency of the vapor pressure on the temperature, a drop to very low temperatures (in soil) could have an effect on the measurement. (iv) The investigation was performed in air where the CO_2_ concentration was too low to influence the measurement result. However, in soil litter, and especially in soils, an increased CO_2_ concentration is to be expected by the turnover of organic carbon. As PDMS has a permeability for CO_2_ that is only about one order of magnitude lower than that for water [25,31], it is important for the applicability of the measurement method to prove the influence of varying CO_2_ concentrations on the measurement. Further investigations are therefore necessary to understand/reduce the impact of these influencing variables on the measurement.

Previous investigations show that the interactions between water molecules are stronger than those between water and PDMS, and the low water uptake of PDMS of <0.2% [40] implies a small concentration of water in the polymer. That causes water to form clusters within the polymer that diffuse through it rather than the individual water molecules [35,40,41,42,43]. This paper proves an equivalent of the water activity $a_{w}^{b}=1.1$ for the membrane surface if the membrane is surrounded by liquid water, i.e., an apparent oversaturation of vapor. This indicates that there is a direct uptake of water clusters from the aqueous environment into the membrane, and this must obviously be more efficient than the uptake of individual water molecules from the saturated vapor (where $a_{w}=1$) and the clustering of these molecules within the polymer. To the author’s knowledge, such a phenomenon has not been described in the literature before.

This change in sorption behavior in turn makes it possible to detect the aggregation state in which water is present around the membrane. It should be possible, therefore, to address additional measurement targets with the MHS that was already introduced in 2019 in [25]. In addition to the measurement of the water potential over a wide range based on the RH measurement, this MHS can be used to detect liquid water in the range, where RH is insensitive to the water potential. Thereby, the use of an internal, vapor-saturated reference, which is an integral part of the measurement principle, allows the distinguishing of the new, liquid water-dependent measurement scale from the one used to measure RH. That means that the measurement of the water saturation should be possible, and the dew point for dry (arid) soil should also be possible to detect using the temperature information provided by the purge gas. Thereby, due to the rapid compensation of local gas pressure differences within the membrane tube, the MHS averages arithmetically over the areas of the tube that are exposed to liquid water or its vapor phase in air. This results in a spatially averaged measure of the water saturation where the averaging length can be preset with the length of the membrane tube in the range of up to several meters. With respect to a point-based dew point detector, such a comparatively large tube-based measurement system could allow specifying, e.g., a spatial condensation probability (i.e., a probability of dew point formation) in a heterogeneous environment.

This present study proves a standard deviation of the measured saturation of about 4% for 1 m long measurement probes (membrane tubes). The response time of the measurement that depends on the properties of the membrane rather than the outer moisture content was in the range of minutes. The new measurement concept can form, therefore, the basis for a novel measurement technique that could achieve acceptable measurement accuracy and temporal resolution. Such a multi-scale measurement technique could be installed directly along a well-defined installation depth in soil or soil litter to, for example, determine the soil moisture, control irrigation, or predict the risk of ignition. This could help to reduce the influence of two important sources of error in soil water measurements with the current direct-measurement sensors [18]: (i) the supporting volume of soil used for the measurement seems to be too small in view of a soil structure that develops dynamically around the sensor. (ii) There is so far no possibility for an in situ calibration of the sensors installed in the soil. However, further investigations will be necessary to achieve this, targeting the respective applications with a technically improved setup, including independent reference measurements.

## 6. Conclusions

This study shows that the response of a PDMS membrane-based cell is dependent on the aggregate state (liquid or gaseous) of the water in its surroundings, even if the chemical potential of the water is the same. It proved that this phenomenon could not be explained by the presence of a diffusive depletion zone in the membrane surroundings but must be based on a mechanism that alters the sorption behavior of the membrane. Thus, an MHS can be used to detect the aggregation state of water, which is not a trivial task when the relative humidity is close to 100%.

This creates a novel basis for spatially averaging moisture measurement, e.g., in heterogeneous soils or soil litter whose applicability potentially extends over the entire moisture range expected in soils. The concept appears to be particularly suitable for long-term use and benefits from a permanent calibration of the measuring system by means of the internal reference measurement that is part of the measurement concept.

Under which conditions (climate, soil types, moisture and temperature range…) and for which applications (moisture monitoring, irrigation control, dew point detection, prediction of forest fire risk…), the measurement technique seems promising has to be clarified in further investigations.

## Figures and Tables

**Figure A1:**
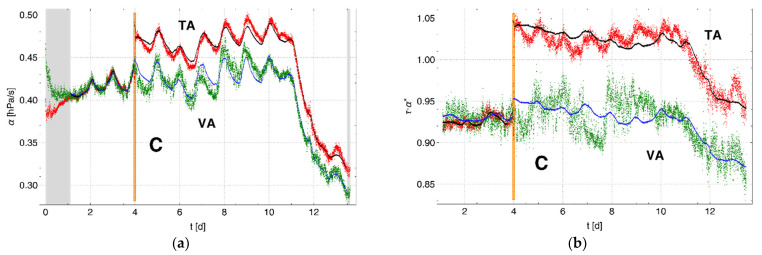
(**a**) Pressure changes analyzed in the test container $\propto_{\mathrm{TA}}$ (red) and vapor container $\propto_{\mathrm{VA}}$ (green) versus the air container. Gray regions represent data that were not included in the further investigation. Data corrected using Equation (12) are shown in (**b**). The solid lines (TA: black, VA: blue) represent calculated pressure changes. The orange box marked C indicates the change from Phase 1 to Phase 2.

**Figure A2:**
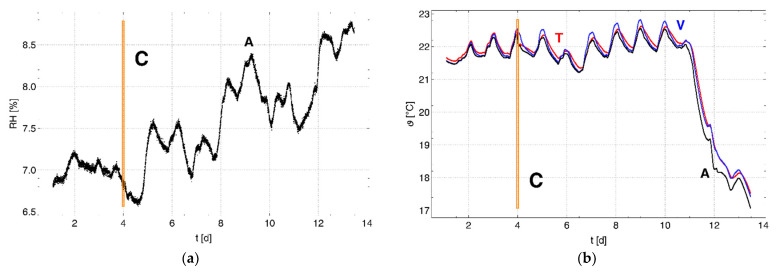
(**a**) Relative humidity in the air container and (**b**) temperatures in the containers (T: red, V: blue, A: black) of the test system. The orange box marked C indicates the change from Phase 1 to Phase 2.

**Figure A3:**
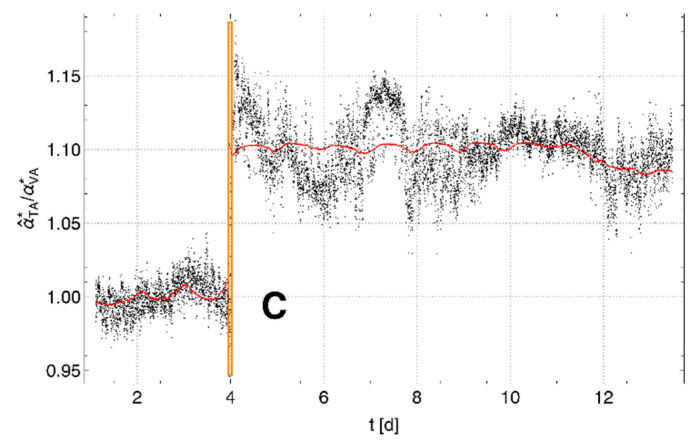
Ratio ${\hat{\alpha }}_{\mathrm{TA}}^{*}/\alpha_{\mathrm{VA}}^{*}$ (black) of the adjusted set of cells for both phases of the experiment. The orange box marked C indicates the change from Phase 1 to Phase 2. The solid lines (red) represent the ratio based on the calculated data.

**Figure A4:**
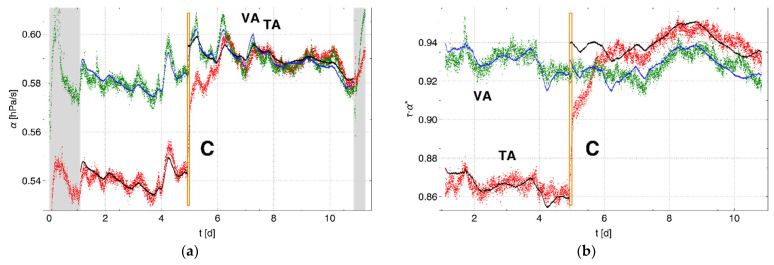
(**a**) Pressure changes analyzed for the test container (TA: red) and vapor container (VA: green) versus the air container. Gray regions represent data that were not included in further investigations. Data corrected using Equation (12) are shown in (**b**). The solid lines (TA: black, VA: blue) represent the calculated pressure changes. The orange box marked C indicates the change from Phase 1 to Phase 2.

**Figure A5:**
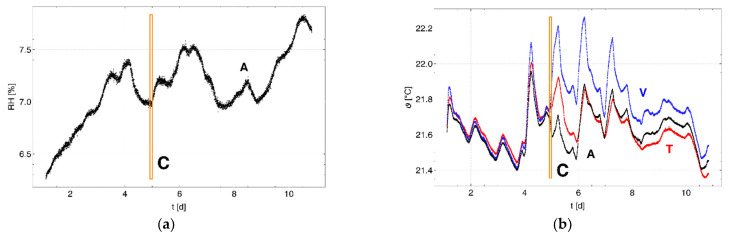
(**a**) Relative humidity in the air container and (**b**) temperatures in the containers (T: red, V: blue, A: black) of the test system. The orange box marked C indicates the change from Phase 1 to Phase 2.

## Data Availability

The corresponding author will be delighted to provide the experimental data on which this study is based on request.

## Appendix A. Experiment 1

**Table A1:** Means (µ) and standard deviations (s) for Experiment 1.

Parameter	Unit	Location	Phase 1		Phase 2	
			µ	s	µ	s
α	hPa/s	TA	0.640	0.002	0.710	0.006
		VA	0.620	0.003	0.650	0.007
ϑ	°C	T	22.52	0.04	22.99	0.17
		V	22.63	0.04	23.26	0.19
		A	22.37	0.04	22.90	0.15
RH	%	T	100.0	<0.1	100.0	<0.1
		V	100.0	<0.1	100.0	<0.1
		A	11.6	<0.1	11.9	0.3
$p_{air}$	hPa	Lab	1017	2	999	5
$p_{in}-p_{out}$	hPa		20.03	0.03	20.11	0.03
*n*	-		1041		1311	

(Lab—measured in the laboratory, *n*—number of data).

**Table A2:** Fit parameters for the linear model $\alpha (\Delta e_{w}^{\prime })$ according to Equation (22) for Experiment 1, fitted to the measurements made in the various containers and experimental phases (δ—standard error, s—standard deviation of residuals, *R*^2^—squared correlation coefficient, n—number of data).

Containers	Phase	$a_{0}\;[\mathrm{hPa}/s]$	$\delta a_{0}\;[\mathrm{hPa}/s]$	$a_{1}\;\left[s^{-1}\right]$	$\delta a_{1}\;\left[s^{-1}\right]$	s [hPa/s]	$R^{2}$	n
TA	1	0.213	0.027	0.0141	0.0010	0.002	0.195	1041
VA		0.171	0.028	0.0147	0.0010	0.003	0.194	
TA	2	0.203	0.005	0.0163	0.0001	0.002	0.901	1311
VA		0.185	0.005	0.0146	0.0001	0.002	0.885	

## Appendix B. Experiment 2

Figure A1a shows the analyzed pressure changes in the test container ($\alpha_{\mathrm{TA}}$, red) and the vapor container ($\alpha_{\mathrm{VA}}$, green) for Experiment 2. Figure A1b shows the pressure changes corrected using Equation (12) and multiplied by the time parameter τ=68.1 s according to Equation (10). The subset of data used for further consideration was chosen between 1.1 and 13.5 d. The means and standard deviations of the measured data for each phase are given in Table A3; the fit parameters for $\alpha (\Delta e_{w}^{\prime })$, according to Equation (22), are shown in Table A4.

**Table A3:** Means (µ) and standard deviations (s) for Experiment 2.

Parameter	Unit	Location	Phase 1		Phase 2	
			µ	s	µ	s
α	hPa/s	TA	0.416	0.009	0.444	0.049
		VA	0.415	0.008	0.405	0.044
ϑ	°C	T	21.91	0.24	21.31	1.44
		V	21.83	0.25	21.29	1.45
		A	21.79	0.22	21.10	1.54
RH	%	T	99.9	<0.1	99.9	<0.1
		V	99.9	<0.1	99.9	<0.1
		A	7.0	<0.1	7.7	0.5
$p_{air}$	hPa	Lab	1015	3	1027	7
$p_{in}-p_{out}$	hPa		20.2	<0.1	20.2	<0.1
*n*	-		2050		6751	

(Lab—measured in the laboratory, *n*—number of data).

**Table A4:** Fit parameters for the linear model $\alpha (\Delta e_{w}^{\prime })$ according to Equation (22) for Experiment 2, fitted to the measurements taken in the various containers and experimental phases (δ—standard error, s—standard deviation of residuals, *R*^2^—squared correlation coefficient, n—number of data).

Containers	Phase	$a_{0}\;[\mathrm{hPa}/s]$	$\delta a_{0}\;[\mathrm{hPa}/s]$	$a_{1}\;\left[s^{-1}\right]$	$\delta a_{1}\;\left[s^{-1}\right]$	s [hPa/s]	$R^{2}$	n
TA	1	−0.102	0.003	0.0170	0.0001	0.002	0.935	2050
VA		−0.004	0.005	0.0139	0.0002	0.004	0.745	
TA	2	−0.060	<0.001	0.0173	<0.0001	0.005	0.989	6751
VA		−0.037	0.001	0.0152	<0.0001	0.009	0.960	

The pressure changes determined in the vapor container show increased noise and a slight shift in time with respect to the measurements taken in the test container. The continuous curve for the vapor container indicates that there are no visible effects due to the change from Phase 1 to Phase 2 in this experiment. Hence, the discontinuity found in Experiment 1 arises in this experiment solely as a function of the state of aggregation of the water around the membrane tube in the test container.

Figure A2 shows the varying experimental conditions for Experiment 2. An opened window in the laboratory significantly increased the temperature variability, and hence, the variability in the vapor pressures in the vapor and test containers. Both pressure-change curves in Figure A1a show a clear dependency on this temperature variability.

The pressure changes were adjusted based on the data for Phase 1, as shown in Figure A1b. A comparatively small scaling factor of κ=1.004, compared to the other experiments, proves that the response behaviors of the cells can already be well tuned to each other by their precise setup.

Figure A3 shows the ratio of the pressure changes ${\hat{\alpha }}_{\mathrm{TA}}^{*}/\alpha_{\mathrm{VA}}^{*}$ (black dots) for the adjusted set of cells. The solid lines (red) represent the ratio from the calculated pressure changes according to Equations (22) and (23). A discontinuity is shown between Phases 1 and 2, where the ratio jumps by >9%. Due to the increased thermal inertia of the test container in Phase 2, its temperature variation is damped and delayed in time with respect to that of the vapor container. The remaining temperature differences are mainly responsible for the fluctuations in the ratio ${\hat{\alpha }}_{\mathrm{TA}}^{*}/\alpha_{\mathrm{VA}}^{*}$.

It is notable that the change in temperature shown in Figure A2b, which caused a strong drop in the pressure-change curves in Figure A1, caused no significant change in the ratio ${\hat{\alpha }}_{\mathrm{TA}}^{*}/\alpha_{\mathrm{VA}}^{*}$. Thus, as long as the temperature differences around the membrane tubes of the MHS can be neglected, this ratio clearly distinguishes whether the membrane is surrounded by vapor or water.

## Appendix C. Experiment 3

Figure A4a shows the analyzed pressure changes for the test container ($\alpha_{\mathrm{TA}}$, red) and the vapor container ($\alpha_{\mathrm{VA}}$, green). The subset of data used for further consideration was selected from the region between 1.1 and 10.8 d. The means and standard deviations of the measured data are given in Table A5. The fit parameters for $\alpha (\Delta e_{w}^{\prime })$ according to Equation (22) are shown in Table A6.

Figure A4 shows that the pressure changes found for the test container are (unlike Experiment 1) smaller than those for the vapor container for the air-surrounded membranes in Phase 1. This may be due to the same reasons identified for Experiments 1 and 2, which are associated with differences in the responses of the cells. However, a matrix effect caused by the membrane surroundings (cotton fabric and sand layer) used in Experiment 3 may also be present.

**Table A5:** Means (µ) and standard deviations (s) for Experiment 3.

Parameter	Unit	Location	Phase 1		Phase 2	
			µ	s	µ	s
α	hPa/s	TA	0.541	0.004	0.590	0.004
		VA	0.581	0.005	0.589	0.005
ϑ	°C	T	21.64	0.12	21.61	0.10
		V	21.63	0.14	21.79	0.15
		A	21.60	0.11	21.65	0.09
RH	%	T	99.9	<0.1	99.9	<0.1
		V	99.9	<0.1	99.9	<0.1
		A	6.9	0.3	7.3	0.3
$p_{air}$	hPa	Lab	1000	4	1006	5
$p_{in}-p_{out}$	hPa		20.1	<0.1	20.1	<0.1
*n*	-		2741		3461	

(Lab—measured in the laboratory, *n*—number of data).

**Table A6:** Fit parameters for the linear model $\alpha (\Delta e_{w}^{\prime })$ according to Equation (22) for Experiment 3, fitted to the measurements taken in the various containers and experimental phases (δ—standard error, s—standard deviation of residuals, *R*^2^—squared correlation coefficient, n—number of data).

Container	Phase	$a_{0}\;[\mathrm{hPa}/s]$	$\delta a_{0}\;[\mathrm{hPa}/s]$	$a_{1}\;\left[s^{-1}\right]$	$\delta a_{1}\;\left[s^{-1}\right]$	s [hPa/s]	$R^{2}$	n
TA	1	0.155	0.005	0.0130	0.0002	0.003	0.657	2741
VA		0.217	0.005	0.0122	0.0002	0.003	0.671	
TA	2	0.210	0.006	0.0128	0.0002	0.003	0.595	3461
VA		0.199	0.004	0.0130	0.0001	0.003	0.751	

Figure A5 shows the test conditions. In Phase 1, the temperature in all the containers follows the daily temperature fluctuation in the laboratory almost exactly. Probably due to its position above the test container and the location of its temperature sensor close to the air in the laboratory (i.e., with no intervening layers of water), the vapor container showed a more pronounced variation in temperature when the test container had been filled with water (Phase 2). The pressure-change curves in Figure A4a follow the trend of the temperatures in Figure A5b.
